# The influence of 3 months of physical exercises and verbal stimulation on functional efficiency and use of free time in an older population under institutional care: study protocol for a randomized controlled trial

**DOI:** 10.1186/s13063-017-2114-1

**Published:** 2017-08-11

**Authors:** Agnieszka Wiśniowska-Szurlej, Agnieszka Ćwirlej-Sozańska, Anna Wilmowska-Pietruszyńska, Natalia Milewska, Bernard Sozański

**Affiliations:** 1 Institute of Physiotherapy, Medical Faculty, University of Rzeszow, Warszawska Street, 35-205 Rzeszow, Poland; 2 Centre for Innovative Research in Medical and Natural Sciences, Medical Faculty, University of Rzeszow, Warzywna Street, 35-959 Rzeszow, Poland

**Keywords:** Elderly, Exercises, Long-term care

## Abstract

**Background:**

In recent years, there has been a significant change in the demographics of developed countries, including an increase in the number of older people. This aging population and the associated need for medical care and assistance places great strain on health care systems worldwide. In older populations, improved knowledge and understanding of the relationship between moderate exercise and health might result in greater motivation to engage in such activity; thus improving the overall health of this population. The aim of the proposed project is to assess the degree of improvement in functional performance through physical training with verbal stimulation, as well as the possibility of changing habitual ways of spending free time, in older people under institutional care.

**Methods:**

Study participants aged 65–85 years, who are living a sedentary lifestyle in care homes in Southeast Poland, will be invited to participate in this randomized controlled trial. Those who meet the eligibility criteria and are enrolled in the study will be assigned at random to one of four parallel groups: (1) basic exercises combined with verbal stimulation, (2) basic exercises without verbal stimulation, (3) functional exercise training with verbal stimulation, and (4) functional exercise training without verbal stimulation. Participants will engage in 30-min workouts, twice per week, for 12 weeks. Tests will be done: (1) before exercises, (2) after 12 weeks of exercises, (3) 12 weeks after the end of the exercises, and (4) 24 weeks after the exercises. Primary outcome measures will include the Short Physical Performance Battery (SPPB). Secondary outcomes will include the Physical Activity Scale for the Elderly (PASE), the Timed Up and Go (TUG) test, the 10-Meter Walk test (10MWT), the Back Scratch (BS) test, the Chair Sit and Reach (CSR) test, the Grip Strength (GS), and the Berg Balance Scale (BBS). Other outcomes will include results regarding postural stability from the stabilometric platform and quality of life (SF-36).

**Discussion:**

Our study will help to determine the effectiveness of the training programs, particularly in relation to participants’ motivation to exercise.

**Trial registration:**

The Sri Lanka Clinical Trials Registry, ID: SLCTR/2016/004. Registered on 12 February 2016.

**Electronic supplementary material:**

The online version of this article (doi:10.1186/s13063-017-2114-1) contains supplementary material, which is available to authorized users.

## Background

Population aging is one of the greatest social and economic challenges facing the European Union (EU). Projections foresee a growing number of persons aged 65 years and over, with a particularly rapid increase in the proportion aged 85 years and over. These demographic developments are likely to have a considerable impact on the health care requirements of older people [1]. According to a 2011 report by the Central Statistical Office in Poland, nearly 40% of people older than 70 years of age have problems with basic self-care: more than 1.8 million have difficulty self-washing, and more than 1.6 million have difficulty dressing and undressing independently [2].

Reduced or impaired mobility leads to poorer quality of life among older people living in residential care homes. Almost 90% of people in long-term care have some type of reduced mobility [3], with about 40% of those with dementia losing their ability to walk each year [4]. Immobility contributes to a loss of ability to perform basic and instrumental activities of daily living and an increased risk of falls and health problems [5]. The adverse consequences of immobility and bed rest are well established; however, older residents living in care homes still spend most of their time in wheelchairs or in bed [6].

Physical activity is an effective strategy for delaying and preventing functional limitations associated with aging. If performed regularly, general physical activity becomes easier. According to the European Opinion Research Group, 97.4% of people aged 65 and over do not meet recommendations for physical activity to achieve health benefits [7]. A Polish study, by Kozdroń, demonstrated that only 7% of people aged 60–64 years and only 0.6% of those aged 80 years or older undertake regular physical activity [8]. In the older Polish population, the most popular use of free time is watching television and listening to the radio (30.2%), followed by reading (15.5%), passive recreation (13.1%), religious practices (11.9%), and gardening (8.7%) [9].

Although physical activity is a part of healthy aging, physical activity levels often significantly decrease with age [10]. According to Ingrid et al. [11], in older people, a lack of participation in regular physical activity is associated with a decline in physical functioning and problems with the ability to perform day-to-day activities. Weeks et al. [12] found that the physical activity levels of older residents living in care homes were lower than those living in the community. Chen points out that institutionalized residents often lead a repetitive lifestyle that lacks variety, which imposes restrictions on activity choices [13]. In another study, the wellbeing and subjective assessments of the quality of life of the study participants was demonstrated to depend on the degree of physical activity of the respondents [14].

In the older population, the low level of knowledge and understanding of the relationship between physical activity and its impact on health may be a barrier in itself. According to Allender, older people have no knowledge about the “proper” amount of physical activity for them, and believe that the activities that they perceive as an “activity” are aimed at younger people [15]. Resnick et al. found that seniors spent increased time partaking in physical activity after the implementation of an incentive program and exercises [16]. According to Dishman, the awareness of the benefits of movement translates into long-term involvement in exercise [17]. Knowledge and belief in the health benefits of physical activity appear to be helpful in motivating an older person to take up exercise on a regular basis.

The most effective classical interventions to improve physical functioning consist of physical exercise, through improved balance, strength and walking practice [18]. According to Westcott systematic strength training positively affects the self-esteem and cognitive abilities of older people [19]. As a result, undertaking physical activity reduces the risk of the development of sarcopenia and related adverse changes. Resistance and similar exercises result in improved gait speed [20]. In their systematic review of 901 care home residents, Brett et al. reported physical exercise to have significant positive effects on cognition, agitation, mood, mobility, and functional ability [21]. de Vreede et al. [22] and Zak et al. [23] conclude that functional task training is the best form of physical exercise; however, de Oliveira et al. [24] report 12 weeks of physical training to have a beneficial effect on the functional state of older people.

Despite being the most effective means of promoting participation in physical activity, at present there are no systematic education and exercise programs designed to improve and maintain functional capacity in Polish care homes for older people. The use of simple, inexpensive and widely practicable exercises and models of motivation for older people in care homes could significantly improve their chances of maintaining or prolonging their independence in performing basic everyday activities and maintaining a good quality of life.

### Objectives

The aim of the project is to assess the degree of improvement in functional performance and the possibility of changing habitual ways of spending free time among older people under institutional care, by applying physical training with verbal stimulation.

## Methods

The study protocol has been written in accordance with Standard Protocol Items: Recommendations for Interventional Trials (SPIRIT). A SPIRIT Checklist is provided in Additional file 1.

### Trial design

**Fig. 1:**
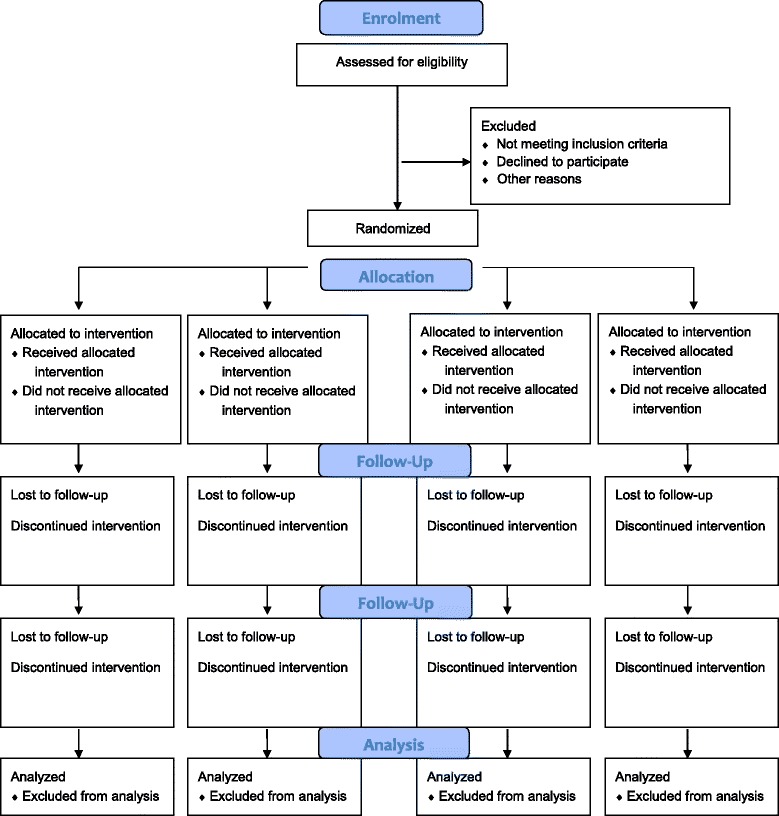
Flow diagram of the study protocol

The research will take the form of a randomized controlled trial with four open-label, parallel groups. Data will be collected at baseline and at 12, 24, and 36 weeks following completion of the program. The Consolidated Standards of Reporting Trials (CONSORT) Statement has been used as a framework for the development of the methodology for this study [25]. The study protocol is illustrated in Fig. 1

### Recruitment

Selected care homes for older people and the chronically physically ill in Southeast Poland will be included in the study. The group of ten nursing homes will be randomly selected. In the case of an insufficient number of participants, another draw will be conducted and the previously chosen residential home will be excluded. Management centers will be informed about the purpose and conduct of the study. After obtaining approval for the project, each center will begin recruiting participants and promotional activities. Pre-qualification for participation in the study will be carried out by a physiotherapist working in the center. A physician employed in a nursing home, after consultation with the research team, will be required to give express written consent to the participation of selected older people in the project, taking into account lack of contraindications to physical exercise. After obtaining written permission from the physician for inclusion in the program, the consent of the study participants prior to baseline assessment will be obtained.

### Participants

Participants eligible for the trial must comply with the following criteria at randomization:

1. Residents of care homes for older people and people with chronic illnesses
2. Aged 65–85 years
3. Mini-Mental State Examination (MMSE) score > 19
4. Geriatric Depression Scale (GDS) score < 20 pkt
5. Spend free time sitting at least 4 h/day, 6 to 7 days/week (Physical Activity Scale for the Elderly (PASE))

Exclusion criteria:

1. Severe systemic disease
2. Severe circulatory or organic insufficiency
3. Severe neurological disorder
4. Lack of consent by the older person or their physician

### Interventions

The subjects will be assigned, at random, to one of four groups:

- Arm 1: basic exercises combined with verbal stimulation
- Arm 2: basic exercises without verbal stimulation
- Arm 3: functional exercise training with verbal stimulation
- Arm 4: functional exercise training without verbal stimulation

**Fig. 2:**
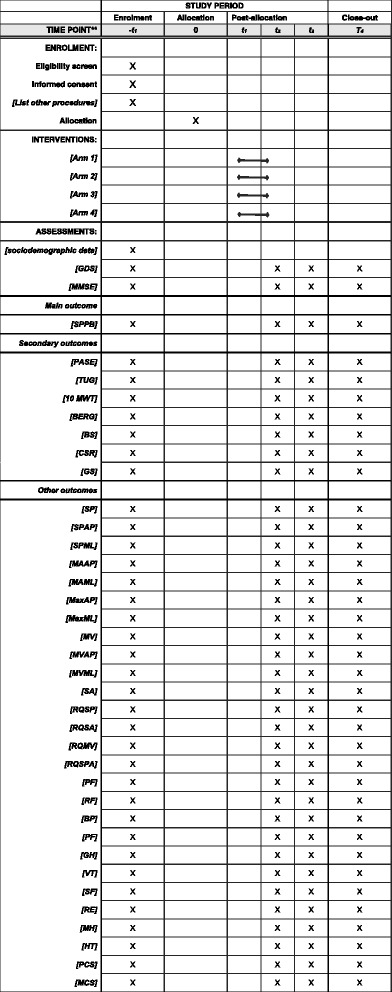
Overall schedule and time commitment for trial participants

Figure 2 shows the overall schedule and time commitment for trial participants.

### Exercise program

#### Basic exercises

The program will include exercises performed in a sitting position. Exercises will contain elements of aerobic, stretching, and equivalent exercises.

#### Functional exercise program

Training will be divided into two sessions. Session I will involve strengthening and stretching, with exercises performed in a sitting position using a Thera-Band and gymnastic sticks. Session II will involve equivalent and functional training using exercises performed in a seated or standing position, using chairs as stabilizing aids. The exercises will contain complex movements that older people perform as a regular part of everyday activity. A single “Session I” and a single “Session II” will take place each week.

The exercise program will be conducted in groups of four to eight, in the care setting, by a qualified physiotherapist. Frequency of training: twice a week for 30 min. Intensity of effort on the Borg Scale: 11–13 points. The frequency of training was determined based on a review by Liu et al. [26]; the intensity of exercise (moderate) was selected in accordance with Canada’s Physical Activity Guidelines [27]. Before and after each workout, blood pressure will be taken and heart rate will be recorded.

#### Verbal stimulation

It is anticipated that verbal stimulation will increase participants’ motivation to undertake physical activity. Presented herein is a model physical exercise program that incorporates the use of verbal stimulation. The model is focused on the study participant’s achievement of (objective) functional goals, and altering their (subjective) perception of the intensity of the training program and individual aims. According to the Competence-based Models of Intrinsic Motivations Theory [28], the efficiency of motivation depends on the person’s conviction of the significance of the set goals.

During baseline assessment, the objectives of exercise participation – both short- and long-term – will be defined. Short-term refers to what a person can do within daily exercises (e.g., perform 20 sit-ups to increase the strength of the lower limbs), whereas long-term objectives concern the “ultimate goal” (e.g., improved mobility, ability of unassisted walking to the grocery). Specified objectives will allow individuals to strive to achieve the goals and thus help them to persevere in their new health behaviors [29]. During the exercise program, progress in achieving functional goals will be analyzed. In accordance with the results of the research by Park et al., [30] determining functional objectives is an important motivation factor for older people to take up physical activity. Moreover, Resnick et al. [31] stated that defining the goals of functional activity, along with achieving and evaluating them, influences the improvement of functional status and increased involvement in physical activity.

The verbal stimulation program, which considers the individual tasks related to the physical exercises, will be adapted to the long-term objectives identified at baseline assessment.

Exercises conducted during classes will be focused on short-term objectives and, prospectively, on achieving the main (long-term) goal.

The verbal stimulation model to be implemented in the presented study is based on the research of Park et al. [30], and will include the following:

1. An assessment of the study participant’s perception of their own performance of the functional activity; specifically, whether the participant perceives their starting position, movement, and final position to have followed the correct motion pattern. The participant will be asked: “Do you feel that you performed your task well?” The question is intended to increase internal motivation
2. A qualitative assessment of task performance will consist of the study participant determining the goal of the activity, and answering the question: “Is the quality of the task you fulfilled, good?” This question is intended to increase internal motivation by connecting their achievement with satisfaction
3. An assessment of the perception of one’s (the study participant’s) own functional activity in accordance with their capability of performing specific tasks. The process will consist of determining the degree of difficulty of the task and evaluating whether performance of the task, despite the degree of difficulty, is feasible. If a task is found to be infeasible, it will be divided into simpler parts. Gradation of difficulty will allow the execution of the task and should allow the participant to generate a more positive association with the activity, helping to eliminate anxiety related to undertaking exercise [32]
4. Provision of support for group members. Each group will be encouraged to focus on one purpose: to increase the functional activity of themselves and each other

According to Locke, raising an older person’s need for physical activity may be achieved by increasing awareness of the motion aspects of human movement and discussing anxieties associated with undertaking physical activity [33].

During the exercise program, physiotherapists will discuss any unpleasant sensations associated with the exercise. Older people will be provided with information explaining how they can reduce pain associated with the activity, if experienced (e.g., hot/cold compresses). Seniors will also be guided as to how to develop a realistic attitude toward the adverse effects of intensified exercises (e.g., muscle pain will not cause further damage to bones or joints).

Verbal stimulation is meant to provide positive stimulation to achieve the success associated with the activity and to overcome any fear of the activity [34].

The verbal stimulation program will be included in the individual exercise programs. The physical exercise programs will be carried out by physiotherapists (one therapist at the nursing home) with at least 2 years of experience in exercise training of older people. Each of the therapists will be trained in exercise programs and verbal stimulation before the start of the program. The intervention will be considered complete if the participant attends at least 80% of the sessions [35, 36]. Therapists will take charge of logging and assessing the attendance of older people at the exercise programs.

The interventions described above involve low risk in relation to health and life. For the duration of the examination, each of the leading therapists will monitor the somatic symptoms of the participants, and determine which directly result from the intervention. Each incident will be dealt with individually and recorded in the protocol of adverse events. In the event of health problems, such as pain or malaise caused by excessive effort, the physician supervising the study will decide on participant exclusion.

During the trial, patients will be advised not to take part in the other group physical activities. Monitoring activities will be conducted therapists responsible for group exercises in the nursing home.

### Outcome measures

Sociodemographic information will be collected and will include age, gender, Body Mass Index, marital status, education level, and time spent in residential care. Depressive symptoms of potential study participants will be measured using the GDS [37], version 15 (GDS-15) [38]. Cognitive function will be evaluated, and potential cognitive impairment identified, using the 30-item MMSE [39].

### Main outcome

The Short Physical Performance Battery (SPPB) is used to evaluate physical fitness [40]. Function of the lower limbs will be assessed using: (1) static balance, (2) gait speed, and (3) getting up from a chair. Each of the elements will be scored from 0 to 4. The final result will be the sum of the points from three attempts.

1. To assess static equilibrium, the participant will perform three stands (side-by-side, semi-tandem, and tandem positions) and hold the position for 10 s
2. To assess gait, participants will be asked to walk a distance of 3 m
3. To assess the strength of the lower limbs, the participant will perform the task five times, without using their hands. The total execution time will be recorded

Data will be collected at the following time points: at baseline (before commencement of the intervention), immediately after completion of the intervention, and 12 and 24 weeks after the completion of the intervention.

### Secondary outcomes

The Physical Activity Scale for the Elderly (PASE) is used for the assessment of physical activity of people over the age of 65 years [41]. It includes an evaluation of the way that free time is spent, and the work-related activities or volunteer work conducted within the 7 days prior to the execution of the survey. The scale consists of 12 questions evaluating the frequency and duration of activity that the participant has partaken in during this time (for example, walks, exercise, housework, gardening, and caring for others) [42]. The overall scoring scale is from 0 to 793, with higher scores indicating a higher level of physical activity. Within the framework of the planned research, validation of the Polish version of PASE will be conducted.

The Timed Up and Go (TUG) test [43] is quick, and is widely used in clinical practice for the initial evaluation of the functional capabilities and balance of older people. The TUG test will be performed as follows: the participant will stand (from a sitting position on a chair), walk a distance of 3 m, turn around (180°), walk the 3 m back to the starting position, and resume the sitting position. The final result will be the average time of three attempts. A cognitive element will be introduced in a second test; specifically, the participant will perform the TUG test while attempting to count backwards, in sets of three, from a number determined by the examiner. A TUG score of ≥13.5 s and TUG cog ≥ 15 s will be used to identify individuals’ risk of falling [44]. Before testing, the research therapist will demonstrate the tasks.

The 10-Meter Walk test (10MWT) is used to evaluate walking speed, and the results of this test correlate with functional efficiency in older people [45]. The test will be considered complete once the participant crosses the 10-m mark, which will be indicated by adhesive tape on the floor. Two attempts will be allowed, with the first being used to determine a relaxed gait speed, and the second the fastest possible, but safe, speed.

The Back Stretch (BS) test is used to assess flexibility of the upper limbs [46]. It will be carried out in a standing position. The participants will be directed to take one hand up and over the shoulder and reach down the back, and the other hand behind and reaching up the back, with the intention of meeting the hands in the middle of the back, between the shoulder blades. Flexibility will be determined as the distance (in centimeters) between the middle fingers.

The Chair Sit and Reach (CSR) test is used to assess flexibility of the lower limbs [47]. From the sitting position (in a chair), the participant will be directed to use two hands (one on top of the other) to reach forward towards the flexed toes of the extended leg (keeping the other bent for stability). Flexibility will be determined as the distance (in centimeters) between the toes on the flexed foot of the extended leg and the fingers of the hands.

Grip Strength (GS) will be assessed using a dynamometer (JAMAR PLUS + Hydraulic Hand Dynamometer, Patterson Medical). Grip force is strongly correlated with level of independence in the performance of activities of daily living and cognitive abilities of older people [48]. The test will be conducted in a sitting position with the adducted arm to the torso and elbow flexion elbow to 90°. The participant will be instructed to shake the dynamometer grip as hard as possible and keep this consistent for 3–5 s. The test will be repeated three times, and the final measurement will be the average of the three results, in kilograms-force.

The Berg Balance Scale (BBS) has been developed to evaluate the risk of falls in the senior population [49]. The test evaluates static and dynamic balance across 14 tasks. Each task will be assessed on a scale of 0 to 4, where 0 indicates a complete inability to perform a task and 4 the ability to independently performance a task. A maximum score of 56 points is possible. When the task is complete, the participant will be classified into one of four groups, indicating the degree of fall risk.

### Other outcomes

Static balance will be clinically assessed using the CQ-stab stabilometric platform. The board platform will be set up in parallel, 2 m from the walls of the room, where a tag will be placed to focus vision with open eyes during the trials. The measuring device will be calibrated before each test. The study will consist of two 30-s trials performed with open and closed eyes. The participant will be asked to conduct the test without shoes, in a relaxed standing position, with arms down by the sides.

Statistical analyses will be used to assess the results of parameters describing the COP (center of pressure of the feet) for measurements with eyes open and closed. The following will be assessed:

- SP – length of path of the statokinesiogram calculated for both axes. It defines the path of the COP during the 30-s sample, given in millimeters
- SPAP – antero-posterior length of COP path, in millimeters
- SPML – medio-lateral length of COP path, in millimeters
- MA – mean amplitude of COP from point 0 (radius), in millimeters
- MAAP – mean antero-posterior amplitude of COP from point 0, in millimeters
- MAML – mean medio-lateral amplitude of COP from point 0, in millimeters
- MaxAP – maximal antero-posterior COP projection from point 0, in millimeters
- MaxML – maximal medio-lateral amplitude of COP from point 0, in millimeters
- MV – mean velocity of COP projection in millimeters per second
- MVAP – mean antero-posterior velocity of COP projection in millimeters per second
- MVML – mean medio-lateral velocity of COP projection in millimeters per second
- SA – field of COP during the test, in square millimeters

In addition, the examination will be subject to the parameters describing Romberg’s coefficient [50]. The coefficient is defined by the quotient of the values of the parameters measured with eyes open by the parameters measured with eyes closed. These will be:

- RQSP – Romberg quotient for the length of path of statokinesiogram, which depicts the COP movement during the test in a two-dimensional coordinate system
- RQSA – Romberg quotient for COP field marked by the movement of COP in a two-dimensional coordinate system
- RQMV – Romberg quotient for the COP means velocity in a two-dimensional coordinate system
- RQSPA – Romberg quotient for the COP field quotient to the COP path length

The quality of life assessment questionnaire (SF-36v2) consists of 36 questions and uses an 11-point scale to evaluate functional health and wellbeing, with the final test results providing a psychometric profile of the examined person. Quality of life according to physical health involves an assessment of the following domains: physical functioning – PF, role functioning – RF, body pain – BP, and general health – GH. Quality of life according to mental wellbeing consists of measurements of: vitality – VT, social functioning – SF, emotional role functioning – RE, and mental health – MH. The following domains are also assessed within the scale: health transition – HT, physical component summary – PSC, and mental component summary – MCS [51].

Data will be collected at the following time points: at baseline (before commencement of the intervention), immediately after completion of the intervention, and 12, and 24 weeks after the completion of the intervention.

### Sample size

An a-priori power analysis was conducted to determine the sample size necessary to find statistically significant exercise effects [52]. The sample size calculation is based on the primary outcome and analysis. The sample size was chosen according to the Cohen method, using standard assumptions: 0.05 for significance level, 0.8 for power of test, 0.5 for effect size which accounts, according to Cohen, for medium effect size. For the final analysis, the total participant number will be 156 (39 in each arm). To allow for a 25% dropout rate, 208 senior participants will be recruited.

### Randomization

Randomization will be carried out by stratified sampling using the statistical package R, version 3.2.2. To obtain an equal distribution of senior subjects across the study groups, the allocation will be performed as block randomization. We will use four-in-one blocks. To solve the problem of predicting the next assignment, the allocator will hide the block size from the executer and use randomly mixed block sizes. Randomization order will be determined using a computer-generated random number schedule. Randomization will be performed by a biostatistician not participating in the study. This person will not participate in the conduct of the research, nor will they be involved in the implementation of the exercise programs. The same person will be responsible for ensuring that the participant list remains confidential. Staff performing outcome measurement and data analysis for the primary outcomes will be blinded to group allocation. However, due to the group nature of the care homes, it will not be possible to blind the participants.

### Statistical methods

The basic characteristics of the groups will be established, and the means and medians will be calculated. The confidence intervals for the mean of quantitative variables will also be determined. Percentage distribution and the number of cases will be established for qualitative variables. To check the differences between the groups, an analysis of variance (ANOVA) will be used for normally distributed variables and a Kruskal-Wallis test for non-normally distributed variables. Normality will be assessed with a Shapiro-Wilk test. We will use the Bonferroni correction to appropriately adjust the overall level of significance for multiple comparisons. Alpha levels will be 0.05 and 0.01.

Basic interactions between the studied groups will also be explored. If there is no interaction, we will examine the independent effects of intervention. If there is significant interaction, the values of the coefficients in the multiple regression model will be tested. Interpretation of research results will be based on estimation and associated 95% confidence intervals.

The analysis will be performed on the ITT set. The analysis will be repeated per-protocol (PP) (in which participants who violate the protocol are excluded from the analysis set) and compared to the ITT analysis as a sensitivity analysis. The multiple imputation (MI) technique will used for dealing with missing data under the assumption that data are missing at random [53].

### Data monitoring

A Data Monitoring Committee (DMC) will be established in cooperation with biostatisticians of the research unit. The DMC will identify the potential need for adjustments. Data will be collected on the cards traditionally used in clinical observation (Case Report Forms; CRFs). In order to ensure the accuracy of the input data, set up will be by two independent individuals who will be responsible for completion of the spreadsheet. The study authors will be responsible for ensuring appropriate storage conditions for the clinical trial.

## Discussion

The International Association of Gerontology and Geriatrics-Global Aging Research Network (IAGG-GARN) and the IAGG European Region Clinical Section has developed recommendations for physical activity for older people receiving long-term care [54]. These recommendations stress the importance of motivation and enjoyment as key factors likely to increase the levels of physical activity in this population. In systematic reviews, de Labra et al. [55] and Cadore et al. [56] demonstrated that, despite published evidence of the positive effects of exercise on physical fitness and quality of life in older populations, comparisons of the effectiveness of various exercise programs were necessary. They stated that such an investigation would provide comprehensive information on the impact of regular physical activity on the functional efficiency and stability of older people. As discussed in detail above, here we propose 12 weeks of intervention followed by 6 months of observation, which should demonstrate the impact of an exercise/motivation program on activity and use of free time in a senior population under institutional care.

Mobility and function are important elements of independent living. For residents of care homes, social assistance can help to maintain residents’ physical, psychological and social function, allowing them to maintain control over their own lives. According to Prata et al., functional autonomy increases satisfaction and enhances the quality of life of older people [57]. Weeks et al. found that preventing the aggravation of functional limitations is another important element in motivating older people to participate in exercise [12]. In the planned study, the authors want to determine which form of physical exercise with verbal stimulation will cause improvement in functional performance and changing habitual ways of spending free time among older people. The results of the study will be used to develop a simple, large-scale exercise program for older adults living in institutional care facilities.

## Trial status

The trial was initiated in March 2016 and is still recruiting participants at the time of submission of the manuscript.

## Additional file

Additional file 1: SPIRIT Checklist. (DOC 121 kb)

## Abbreviations

10MWT: 10-Meter Walk test
BBS: Berg Balance Scale
BP: Body pain
BS: Back Scratch
COP: Center of pressure
CSR: Chair Sit and Reach
DMC: Data Monitoring Committee
EU: European Union
GDS: Geriatric Depression Scale
GH: General health
GS: Grip Strength
HT: Health transition
MA: Mean amplitude of COP from point 0 (radius)
MAAP: Mean antero-posterior amplitude of COP from point 0
MAML: Mean medio-lateral amplitude of COP from point 0
MaxAP: Maximal antero-posterior COP projection from point 0
MaxML: Maximal medio-lateral amplitude of COP from point 0
MH: Mental health
MMSE: Mini-Mental State Examination
MSC: Mental component summary
MV: Mean velocity of the COP projection in millimeters per second
MVAP: Mean antero-posterior velocity of the COP projection in millimeters per second
MVML: Mean medio-lateral velocity of the COP projection in millimeters per second
PASE: Physical Activity Scale for the Elderly
PF: Physical functioning
PSC: Physical component summary
RE: Emotional role functioning
RF: Role functioning
RQMV: Romberg quotient for the COP means velocity in a two-dimensional coordinate system
RQSA: Romberg quotient for COP field marked by the movement of COP in a two-dimensional coordinate system
RQSP: Romberg quotient for the length of path of statokinesiogram, which depicts the COP movement during the test in a two-dimensional coordinate system
RQSPA: Romberg quotient for the COP field quotient to the COP path length
SA: Field of COP during the test, in square millimeters
SF: Social functioning
SP: Length of path of the statokinesiogram calculated for both axes. It defines the path of COP during the 30-s sample
SPAP: Antero-posterior length of COP path
SPML: Medio-lateral length of COP path
SPPB: Short Physical Performance Battery
TUG: Timed Up and Go
TUG cog: Timed Up and Go Cognitive
VT: Vitality
